# Developing a structured framework to explore the experiences of people with dementia and their caregivers regarding non‐pharmacological sleep interventions

**DOI:** 10.1002/alz.71081

**Published:** 2026-02-07

**Authors:** CAM Huisman, MGLC Loomans, HSM Kort

**Affiliations:** ^1^ Research Group Technology for Healthcare Innovations Research Centre Healthy and Sustainable Living University of Applied Science Utrecht Utrecht The Netherlands; ^2^ Department of the Built Environment Building Lighting group, Health in the Built Environment, Eindhoven University of Technology Eindhoven The Netherlands; ^3^ Department of the Built Environment, Building Performance group, IEQ‐Health Eindhoven University of Technology Eindhoven The Netherlands

**Keywords:** aging‐in‐place, cognitive impairment, elderly care, indoor environmental quality, informal care, sleep interventions, structured framework

## Abstract

**INTRODUCTION:**

This paper presents the development of a framework to assess the use and experiences of non‐pharmacological interventions (NPIs) supporting sleep, including technological and indoor environmental quality (IEQ) measures. Sleep disturbances are common in people with dementia (PwD) and increase caregiver burden. Pharmacological treatments pose risks, highlighting the need for effective NPIs.

**METHODS:**

The framework was designed through literature review and expert consensus, and piloted over three weeks with two community‐dwelling PwD and one caregiver.

**RESULTS:**

Findings were analyzed to improve the framework on explanation of NPIs, questionnaires, and sleep monitoring. The framework integrates methods to assess user experiences and to monitor sleep and IEQ parameters, due to their impact on sleep.

**DISCUSSION:**

The final framework, DESMEE‐CAP, has demonstrated validity and utility in capturing experiences without disrupting routines. While promising, the small sample size limits generalizability.

**Highlights:**

- Development of a framework, in co‐creation, that supports research on the use of non‐pharmacological interventions (NPIs) for sleep support for community‐living people with dementia and their caregivers.
- Attention to sleep quality and appropriate support is needed, and insights are provided through the use of the developed framework.
- Contribution to the development of appropriate, non‐pharmacological support for sleep of people with dementia and their caregivers at home.

## 1 BACKGROUND

Sleep disturbances are common in people with dementia (PwD).^1^ Nevertheless, most PwD live in the community, supported by informal caregivers, such as family members, friends, or neighbors,^2^ as PwD generally prefer to remain at home for as long as possible.

Sleep disturbances affecting approximately 70% of the PwD.^1^ Sleep disturbances also impact caregivers,^3^ increasing their care burden and often contributing to the transition of PwD to a long‐term care facility.^4^, ^5^ Sleep support may alleviate these figures. Brown et al.^6^ emphasized that such support should address both nocturnal sleep and daytime functioning and consider the needs of both PwD and their caregivers.

Based on the literature discussed below, a suit of intervention options to address sleep quality issues. In general intervention options relate to pharmacological and non‐pharmacological interventions (NPIs). The focus of this research is on NPIs. NPIs comprise technology‐based and indoor environmental quality (IEQ) ‐related interventions. IEQ comprises factors as light, air quality, thermal, and sound/ acoustical conditions. Additionally, the coping strategies of PwD and their caregivers should be considered.^6^ Brown et al.^6^ found that strategies such as going to bed together and engaging in intellectual and physical activities during the day promote better sleep.

Pharmacological interventions are often used to address sleep issues^7^; however, they are not always effective^8^ and may pose risks for PwD due to sedative effects, which can increase risk of falls and exacerbate cognitive decline.^9^ Alternatively, NPIs have shown promising outcomes in supporting sleep.^10^ In this context, NPIs include natural remedies, traditional medicine, complementary treatments, supportive care, lifestyle modifications, and wellness practices,^11^ but also light therapy.^12^

Apart from the mentioned examples, NPIs may also involve coaching, technological solutions, and modifications to the built environment, including the IEQ. Several studies showed the effect of IEQ parameters on sleep quality. Basner et al. (2023) concluded that temperature, noise, particulate matter (PM2.5), and CO_2_ levels in the bedroom affect sleep efficiency.^13^ Buonanno et al. (2024) found that higher CO_2_, relative humidity (RH), and temperature levels decrease sleep quality in good sleepers.^14^ The IEQ when it concerns light, sound, and temperature, has shown to affect PwD admitted to a neuropsychiatric ward resulting in verbal and/or motor agitation.^15^ In this observational study on a neurogeriatric ward, agitation is predicted to occur due to IEQ parameters within a short period of time (approximately 20 min).^15^ More studies have examined the effects of IEQ in a single variable approach.^16^, ^17^, ^18^ The research context of these studies was though not the home setting of PwD. Technological interventions are increasingly recognized for their potential to enhance the lives of PwD.^19^ Although, there is a growing need to develop these technologies for application outside of long‐term care settings.^20^ While Kort et al.^21^ demonstrated that PwD and their caregivers can be actively involved in research, a review by Øksnebjerg et al.^22^ identified a lack of well‐designed studies on assistive technologies in this population.

**RESEARCH IN CONTEXT**

1. **Systematic review**: The authors reviewed literature using traditional sources and relevant conference abstracts. Testing non‐pharmacological interventions in the homes of people with dementia has not yet been widely studied. The same applies to the influence of indoor environment quality on people with dementia. Nevertheless, there are relevant publications regarding these topics. These publications are appropriately cited in our manuscript.
2. **Interpretation**: This study aims to explore the experiences of people with dementia and their caregivers with non‐pharmacological interventions—both technology‐based and built environment‐related—to assess sleep support by using a framework called DESMEE‐CAP. The findings demonstrate the frameworks usability in this context, based on a pilot study.
3. **Future directions**: With some modifications, the framework can be flexibly used to assess sleep support, allowing researchers to monitor sleep and indoor environmental quality. Using this framework may contribute to the developing appropriate sleep support for people with dementia and their caregivers.

Moreover, the World Health Organization^20^ recommends conducting dementia research in settings that closely resemble real‐life environments. They also advocate for a shift in focus toward outcomes such as improved quality of life, social health, and enhanced daily functioning. These recommendations emphasize the importance of developing and testing technology in collaboration with care professionals, engineers, PwD, and their caregivers. This also highlights the need for guidelines and evidence‐based methods to support and formalize user involvement.^22^ To evaluate the usability of new and existing technologies, it is essential to assess them in practice with their potential end‐users. In order to better compare NPIs’ strategies, a framework should be available that enables analysis of NPIs, comprising both IEQ based as well as technological based interventions.

In the context of sleep problems in PwD, the study of Huisman et al. (2025)^23^ identified three key themes related to sleep in PwD and their caregiver. Sleep problems may arise due to (a) challenges in maintaining time orientation and day/night 24 routines, (b) irregularities and concerns of informal caregivers at night, and (c) environmental cues that either support or disturb sleep.^23^

Returning to the importance of the IEQ, Richards et al. (2004) mention that a mismatch between person's needs and abilities with the environment may lead to insufficient sleep which can trigger behavioral symptoms in PwD.^24^ These symptoms are also addressed as displaying challenging behaviour. Apart from that, IEQ is known to influence sleep quality in healthy (older) people.^25^, ^26^, ^27^ For example, relationships are found between sleep and indoor air quality (IAQ), light exposure, and thermal environment.^28^, ^29^ Although, the research populations from these studies do not resemble PwD or their caregivers.

Also, Canha et al.^30^ highlighted the importance of assessing sleep quality to better understand the impact of indoor environmental factors. As research in this field, specifically focusing on community living PwD and their caregivers, is virtually non‐existent, there is a need to explore the extent to which IEQ factors affect the sleep of PwD. This to better value its potential as a NPI option, alongside other NPI alternatives.

Summarizing, this study aims to develop a structured framework to gain insights into the experiences of community living PwD and their caregivers with the use of NPIs technology and IEQ related. A better understanding of the potential IEQ conditions and other NPIs may have on sleep issues experienced by both PwD and their caregivers is essential for developing effective interventions.

In the next section, the development of the framework is described, followed by the description of the framework. Additionally, the framework is used in a pilot study to test its functionality to capture the experiences of PwD and their informal caregivers. The findings of this study will be discussed and provide adjustments to the initially developed framework.

## 2 METHODS

**FIGURE 1:**
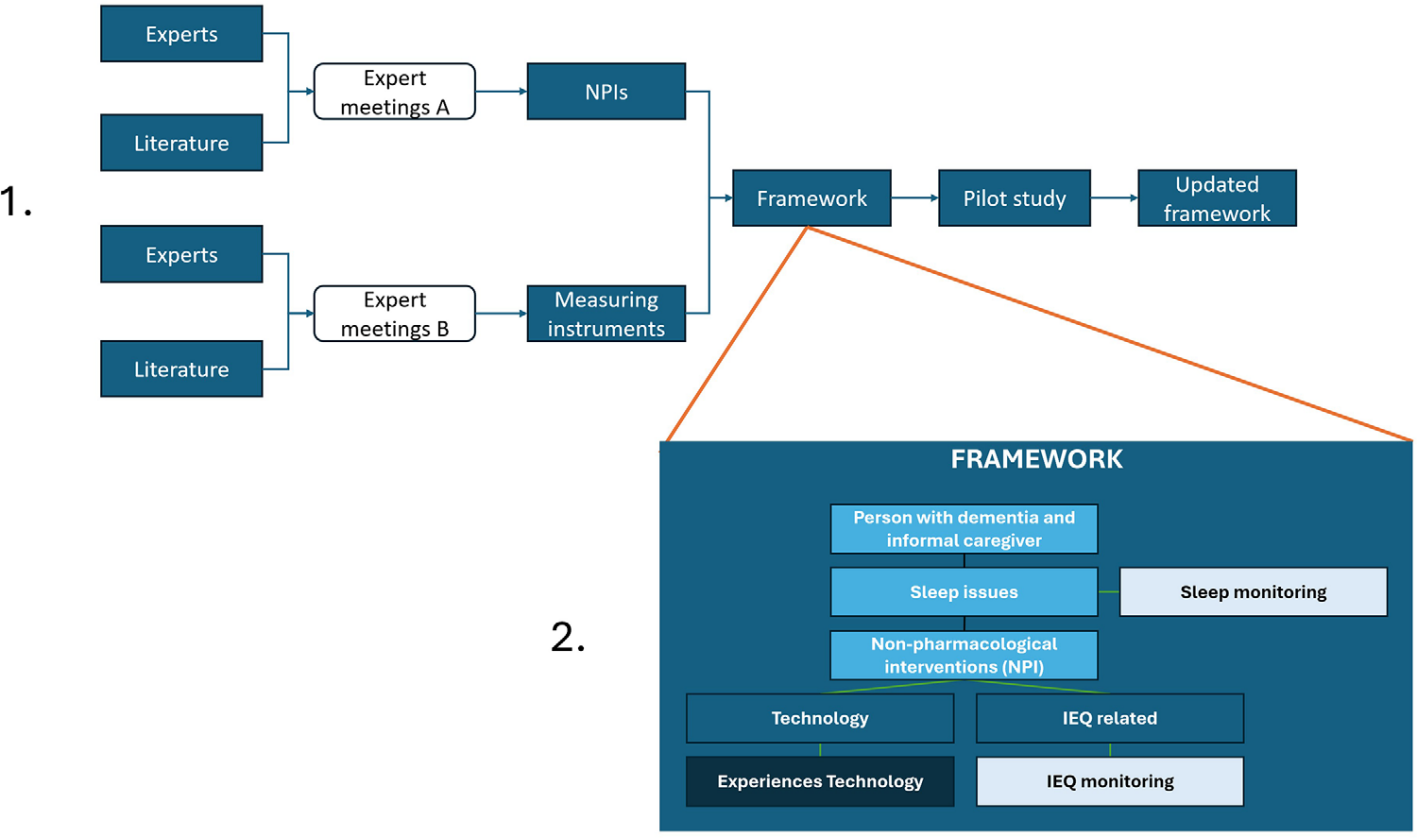
(1) Development steps of the framework and (2) the building blocks of the DESMEE‐CAP framework

The framework is developed with experts who shared their expertise in several expert meetings in which via iteration and triangulation two directions are taken. One focusing on NPIs selected based on strategies to support sleep and another to measuring instruments to explore use and experiences and to monitor sleep and IEQ (Figure 1‐part 1). Figure 1 (part 2) shows the building blocks of the framework. Each building block is discussed under paragraph 2.2 DESMEE‐CAP Framework.

### 2.1 Experts and literature

For the design of the framework, experts were consulted, and relevant literature was reviewed for triangulation. A convenience sample of seven experts from the Netherlands is selected. The experts were representatives of the dementia field; specializing in dementia, user research, indoor environment, implementation, and innovation in healthcare and wellbeing.

In the expert meetings, four strategies to promote sleep in PwD and their caregivers are presented based on the themes identified by Huisman et al. (2025).^23^ During two iterative expert meetings (Figure 1 meetings A), in line with these strategies an overview of technological interventions is presented and potentially successful NPIs are discussed and selected. During the meeting consensus was reached which NPI matches with the presented strategies as shown in Table 1.

**TABLE 1:** Overview of the NPIs with the aligned themes and strategies

Theme (based on Huisman et al. (2025))	Strategy (based on Huisman et al. (2025))	NPI / product	Characteristics
Maintaining time orientation and routines Irregularities and concerns of the informal caregiver Environmental cues	Support maintaining a daily rhythm Support the transition from day to night	*Lizz sleep coach*	‐ Stand‐alone tablet with faceplate ‐ Paired with caregiver (in development) ‐ Agenda with visual and spoken reminders ‐ Support transition day to night ‐ Collecting self‐reports ‐ Social interaction
Maintaining time orientation and routines Irregularities and concerns of the informal caregiver Environmental cues	Support maintaining a daily rhythm Support the transition from day to night	*TimeSteps*	‐ Application ‐ Paired with caregiver ‐ Time orientation ‐ Agenda with visual and spoken reminders
Irregularities and concerns of the informal caregiver Environmental cues	Support falling asleep Sleep through	*Somnox 2*	‐ Sleeping aid ‐ Falling asleep
Irregularities and concerns of the informal caregiver Environmental cues	Support falling asleep Sleep through	*Qwiek.snooze*	‐ Music pillow ‐ Falling asleep ‐ Sleeping through
Irregularities and concerns of the informal caregiver Environmental cues	Support falling asleep Sleep through	*Weighted blanket*	‐ Blanket ‐ Falling asleep ‐ Sleeping through

Abbreviation: NPIs, non‐pharmacological interventions.

To be able to select appropriate NPIs it is necessary to gain better understanding regarding the home situation, way of life, and preferences of the users. So, besides the NPI selection, introduction questions are discussed for an appropriate selection of NPIs (see Personal selection of technology NPI).

After the consensus about relevant NPIs that the framework should be able to investigate, an analysis of required performance indicators and related measuring instruments was performed, again relying on literature and expert meetings (B) (Figure 1). This analysis was focused on two distinct components: (1) gathering lived experiences of using non‐pharmacological (NP), technology‐based interventions and (2) monitoring IEQ and sleep.

During two iterative expert meetings indicators and methods to gain experiences of the users were discussed, while input from a literature search conducted in Google Scholar using the keywords dementia, IEQ, sleep, monitoring, technology, and NP interventions and the protocol of Mishra,^21^, ^22^ was used. The experts assessed the feasibility of monitoring sleep and IEQ in community‐dwelling people with dementia and their caregivers. Important aspects discussed during the meeting were that all research methods should be easy to use, and are not too demanding for the target group. The outcomes of expert meetings A and B were inductive combined, resulting in the framework named DESMEE‐CAP.

### 2.2 DESMEE‐CAP framework

**FIGURE 2:**
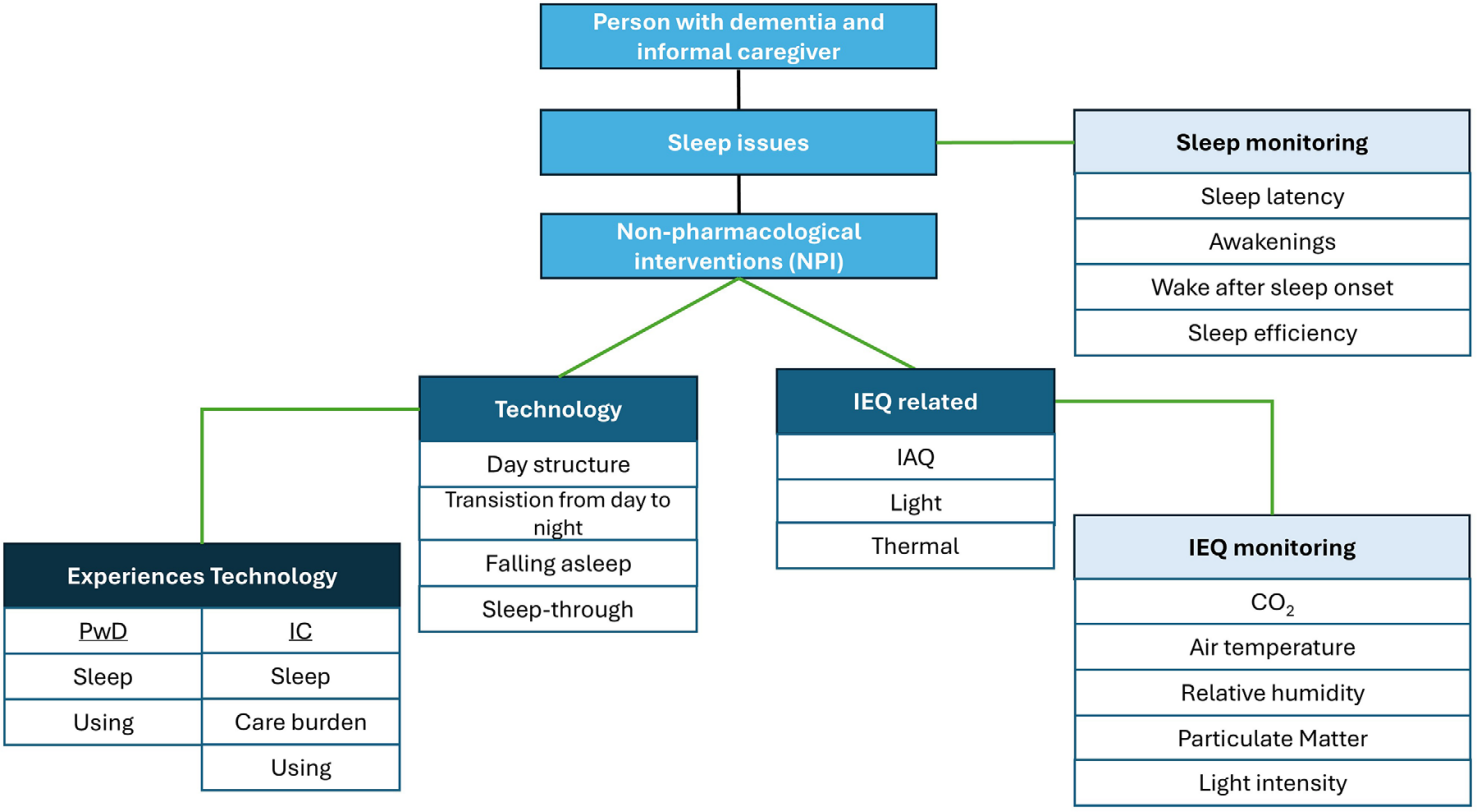
Framework of the DESMEE‐CAP addressing sleep issues of PwD and their caregivers and NPI components Technology‐related or IEQ‐related and respectively the measurements to capture the experiences and to monitor the IEQ and sleep. The green lines represent the connection between the components of the framework. IAQ, indoor air quality; IC, informal caregiver; IEQ,; PwD, people with dementia

The target group may benefit from NPIs that are either technology‐based or IEQ‐related. Central to this framework are the experiences of PwD and their informal caregivers with the technology‐based NPIs. The IEQ‐related type of NPI is monitored to gain deeper insight into environmental factors that may influence sleep. In Figure 2 the framework of the DESMEE‐CAP is presented. The individual parts of the framework are discussed below.

#### 2.2.1 People‐context

The framework starts with the PwD and their caregivers as the target group. The focus is on community‐dwelling people with dementia and their caregivers who experience sleep‐related issues. Based on the expert meetings the following inclusion criteria are determined: (A) the person with dementia must be able to participate in a conversation involving both the researcher and the informal caregiver; and (B) at least one participant has a smartphone. The exclusion criteria are: (1) individuals in an advanced stage of dementia, and (2) individuals currently receiving treatment for sleep problems (e.g., therapy or medication).

#### 2.2.2 Sleep issues

The overarching objective is to facilitate sleep in PwD and their caregivers, as sleep issues impact both. Sleep issues in PwD are for example short sleep duration, fragmented sleep, altered circadian rest/activity patterns, and an increase of sleep disordered breathing^31^

#### 2.2.3 NPIs

The NPIs comprise both IEQ‐related and technology‐based interventions. The latter are described in Table 1 with details in Appendix 2. Based on expert opinion, a maximum of two interventions per household is recommended to avoid overwhelming or overburdening participants.

Five NP technology‐based interventions illustrated in Figure 2 under technology were selected. These interventions correspond to themes identified in the study by Huisman et al. (2025)^23^, which explored sleep in PwD living at home. The three themes identified were: (a) maintaining time orientation and routines, (b) irregularities and concerns of informal caregivers, and (c) environmental cues.^23^ These themes align with four strategies: (1) support maintaining a daily rhythm, (2) support the transition from day to night, (3) support falling asleep, and (4) sleep through (see Table 1). These strategies are also based on a previous study.^23^

The selected NPIs that can be handled with the framework include the Lizz sleep coach, the TimeSteps app, the Somnox sleeping aid, Qwiek.snooze music pillow, and a weighted blanket. Some details of these NPIs are provided in Table 1, third and fourth column and in Appendix 2. As can be seen in Table 1 theme (a) Maintaining time orientation and routines is addressed through strategies 1 and 2. Theme (b) Irregularities and concerns of the informal caregiver refers to strategies 2 and 4. While, theme (c) Environmental cues comprises all strategies.

The IEQ‐related NPIs illustrated in Figure 2 include IAQ, indoor lighting, and the thermal environment. These IEQ parameters were selected because they appear to affect people's sleep.^15^, ^24^, ^25^, ^26^, ^27^, ^28^, ^29^, ^32^

#### 2.2.4 Personal selection of technology NPI

As perception of the NPI is an important parameter in understanding its success, it is important to understand how the selected NP technology‐based intervention is received by the PwD and their caregiver. For a sensible analysis of an NPI technology, it is important to select technologies, per case, which are well suited to the user. For example, it is essential that both the person with dementia and their caregiver are able to familiarize with the specific NPI. Therefore, the following questions (see Table 2) are composed together with the experts to gain a better understanding regarding the home situation, way of life, and preferences of the PwD and caregivers. Furthermore, the responses of the introduction questions (Table 2) and the discussion of potential strategies including NPI, give directions to the researcher, in consultation with the participants, to propose which NPIs can be used. Points of discussion with the PwD and their caregivers include technology skills, problems experiences, and how often someone is away from home. Questions are developed (expert meetings A) and are about the daily living of a dyad, the sleeping conditions, and the problems during day and night.

**TABLE 2:** Introduction questions to support the selection of relevant NPIs for each specific case

Introduction questions	
Main question	Sub question
What does your daily life look like?	How do you spend your days? Are you at home or do you go out a lot?
Describe a normal weekday	Can you describe what a normal day looks like for you? What time do you get up? What are the first things you do? What does your afternoon look like? What time do you go to bed?
Describe a normal weekend	Can you describe what a normal weekend day looks like for you? What time do you get up? What are the first things you do? What does your afternoon look like? What time do you go to bed?
Normal sleeping conditions	What kind of duvet/pillow (synthetic, wool, down, bamboo, cotton) do you use? What kind of sleepwear do you sleep in? Do you sleep with the window open or closed? Do you sleep with the door open or closed?
Bottlenecks during the day	Are there things you experience difficulties with during the day? ‐ For example: activation, finding activities
Bottlenecks at night	Are there things you experience difficulties with in the evening/night? For example: not being able to fall asleep, waking up, wandering

Abbreviation: NPIs, non‐pharmacological interventions.

#### 2.2.5 Experiences with the technology based NPIs

The experience with the NPIs themselves will be monitored as part of the framework. When one or two technology NPIs have been selected evaluation takes place. For that, user experiences are gathered through qualitative measurements (interviews and notebook), during and after the intervention period. In consultation with the experts a semi‐structured interview was developed, with a basis in the Caregiver Strain Index (CSI)^33^ and the USE questionnaire (Usefulness, Satisfaction, and Ease of Use).^34^ The adapted version of the questionnaire ensures that it is appropriate, based on experts’ experiences, for vulnerable older adults.

The CSI^33^ is chosen to assess caregiver strain. The USE questionnaire^34^ captures user experiences with the technology NPIs. The relevant indicators from both instruments were compiled into two topic lists (see Table 3) to guide the semi‐structured interview at the end of the study period. In that interview relevant information about the experiences of PwD and their caregivers with using NPIs to support sleep during the night is collected.

**TABLE 3:** Topic lists to gather experiences with NPIs s and the study

Start question: Have you used the interventions?	
No	Yes
Topic list a	Topic list b
Can you talk a bit about your experiences with the interventions (asked per intervention)? Can you explain why you did not use the intervention(s) or only used it for part of the period? Knowledge and skills, ability to use it Fear Usefulness of the intervention(s) Can you talk a bit about using the intervention(s)? (asked per intervention) Ease of use Comprehensibility Ease of learning Satisfaction Fear of using it? Did you run into any other issues? What would be helpful for you? Can you tell us what you thought about participating in this research? Information / explanation Home visits Accessibility project team Measurement equipment (sensors)	Can you talk a bit about your experiences with the interventions (asked per intervention)? Can you talk a bit about with what the interventions (asked per intervention) helped you? Can you talk a bit about how the interventions (asked per intervention) have helped you? Intervention 1 Intervention 2 Intervention 3 Was the combination of interventions helpful? (if applicable)? 4. Can you talk a bit about what the interventions (asked per intervention) finally gave you? Intervention 1 Intervention 2 Intervention 3 What did the combination of interventions give you? (if applicable) Can you talk a bit about using the interventions? Ease Comprehensibility Ease of learning Satisfaction Fear of using it? Did you run into any other issues? Can you talk a bit about how the technology could be improved for you? (What can be improved?) Intervention 1 Intervention 2 Intervention 3 Could you tell us what you thought about participating in this project/study? Information / explanation Home visits Accessibility project team Measurement equipment (sensors)

Abbreviations: NPIs, non‐pharmacological interventions.

Two topic lists are developed (Table 3): (a) for participants who did not use the NPIs, or only partially used them during the study period (by choice); and (b) for those who used the NPIs. Topic list (a) focuses on understanding why the interventions were not used and what would be needed to encourage future use. It also includes questions about participants’ experiences of taking part in the study. Topic list (b) is designed for participants who used the NPIs and explores their experiences, including perceived support, usability (ease of use, understandability, ease of learning, satisfaction, and fear of use), and suggestions for improvement of the technology‐based NPIs. This list also includes questions about their experience of participating in the study.

PwD and their caregivers should have access to a help desk via phone or email for support or to share comments. Additionally, with the help of a notebook they can document anything related to their experiences.

#### 2.2.6 Sleep and IEQ monitoring

The experts suggests that in addition to the experiences with the NPIs, the sleep of participants and IEQ in the bedroom and living room should be monitored as part of the framework.

##### Sleep monitoring

In consultation with the expert group it was decided that the following sleep quality parameters should be monitored: sleep latency, awakenings, wake‐up after sleep onset, and sleep efficiency.^35^ Because of the context, PwD and their caregivers, the consideration is that it is important to use non‐intrusive monitoring techniques (e.g., sensors) to monitor these sleep parameters. In the case of PwD, a ring (Oura ring, generation 3) may be preferred over a bracelet because of the need to wear the sensor at night to monitor sleep quality objectively. The assumption is that a ring is perceived as more normal and less conspicuous.

Besides that, it is decided to perform a subjective measurement of the sleep of informal caregivers by using a questionnaire based on two validated questionnaires, the Groninger Sleep Quality Scale^36^ and four questions from the Morning questionnaire.^37^ An overview of the measurements as part of the framework is shown in Table 4.

**TABLE 4:** Overview of the building blocks in the DESMEE‐CAP framework

Items	Rationale	Based on Mishra et al. or addition
People‐context	Recruitment focused on community living people with dementia and their caregivers, with a smartphone or tablet available. Introduction questions used during the start meeting of the research.	Added
Conditions	Participants continue their normal everyday lives during the study	Mishra et al.
**Measuring instruments**		
Experiences		
Experiences with NPIs	Semi‐structured interview at the end of the study period. Topics based on the Caregiver Strain Index ^33^ and USE questionnaire ^34^. The CSI is focused on the care burden of the informal caregiver. The USE questionnaire focuses on the usability of products and services. A notebook is available for participants, to take notes regarding the study and/or NPIs. During the research period a helpdesk is available by phone or email for support or to share any comments.	Added
Sleep		
Monitoring sleep subjective	Objective monitoring, to monitor sleep latency, awakenings, wake after sleep onset, and sleep efficiency. Subjective monitoring sleep of the informal caregiver by using a daily morning questionnaire, based on the Groninger Sleep Quality Scale and the Morning questionnaire.	Mishra et al.
Monitoring Sleep objective Oura ring (gen. 3)	Sleep quality objectively monitored using the following parameters: sleep latency, awakenings, wake‐up after sleep onset, and sleep efficiency.	Change of sensor, see paragraph Sleep monitoring
IEQ		
CO_2_ Airteq sensor	Strøm‐Tejsen et al. (2016) conclude in their study that a high CO_2_ concentration leads to a lower experience of air quality during the night. A high CO_2_ concentration could increase Sick Building Syndrome symptoms, for example, eye irritation.	Mishra et al.
Air temperature Airteq sensor	Xu and Lian mention that more needs to be known about the link between thermal comfort and sleep quality. However, others mention that when individuals experience thermal satisfaction or neutral thermal sensation, they have improved sleep quality. Tartarini et al. conclude in their study that variation in air temperature can lead to agitation in nursing home residents with dementia, and a comfortable environment increases well‐being.	Mishra et al.
Relative humidity Airteq sensor	Relative humidity is a parameter affecting the indoor environmental quality. Less is known about the correlation with sleep quality. Nevertheless, in thermal environment temperature and humidity are linked. Besides that, Tsuzuki et al. reported the effects of humidity (out of the acceptable range) on sleep quality and suggested lower sleep efficiency caused by higher humidity.	Mishra et al.
Particulate matter (PM2.5) Airteq sensor	The review of Cao et al. shows that air pollutants, especially PM and NO_2_, are triggers for poor sleep quality. The placement of the sensor is in line with the protocol of Mishra et al.	Added
Light intensity^a)^ AEther sensor	Light exposure affects the circadian rhythm, among other things. A study by Sloane et al. in long‐term care facilities shows that light during the day positively affects residents' sleep. The sensor is placed near the sleeping place of the person with dementia (as far as possible) with the sensor facing the window.	Added

^a)^ The sensor used, developed by Utrecht University of Applied Sciences, measured the presence of light (lux) in qualitative steps (non‐continuous scale).

Abbreviations: IEQ, indoor environmental quality; NPIs, non‐pharmacological interventions.

##### IEQ monitoring

To obtain an indication of the IEQ in the participant's house, several aspects of the IEQ will be monitored. These aspects, including their rationale, are provided in Table 4, with some additions, follow the protocol of Mishra et al.^37^

The protocol used by Mishra et al.^37^ is used for monitoring the IEQ. This protocol is a modification of the protocol developed by Strøm‐Tejsen et al.^25^. Because the protocol was used in other target groups, adjustments were necessary. The IEQ parameters (see Table 4) should be monitored in the bedroom and in the room where the person with dementia spends time during the day (e.g., the living room). This latter monitoring is important because PwD sometimes naps in a chair or couch.^32^ The sensor in the bedroom should be placed near the bed of the person with dementia, ensuring that direct breathing on the sensors is avoided. Given the participants' vulnerability and potential for confusion, placing the sensor(s) in a logical and unobtrusive location is essential. In any case, consent is required from the participants regarding the position of the sensor(s) in the room.

### 2.3 Pilot study setup

In order to test the DESMEE‐CAP framework, a pilot study of an exploratory nature was executed. The pilot study was conducted to qualitatively assess the face validity of the DESMEE‐CAP framework. An important aspect of the study was confirming the practically and usability of the framework in a real‐life setting, with the option to improve the framework based on the experiences from the study. The Health Domain Research Ethics Committee at Utrecht University of Applied Sciences approved the study protocol of the pilot (ECO‐GD, ref. 188‐000‐2022).

**FIGURE 3:**
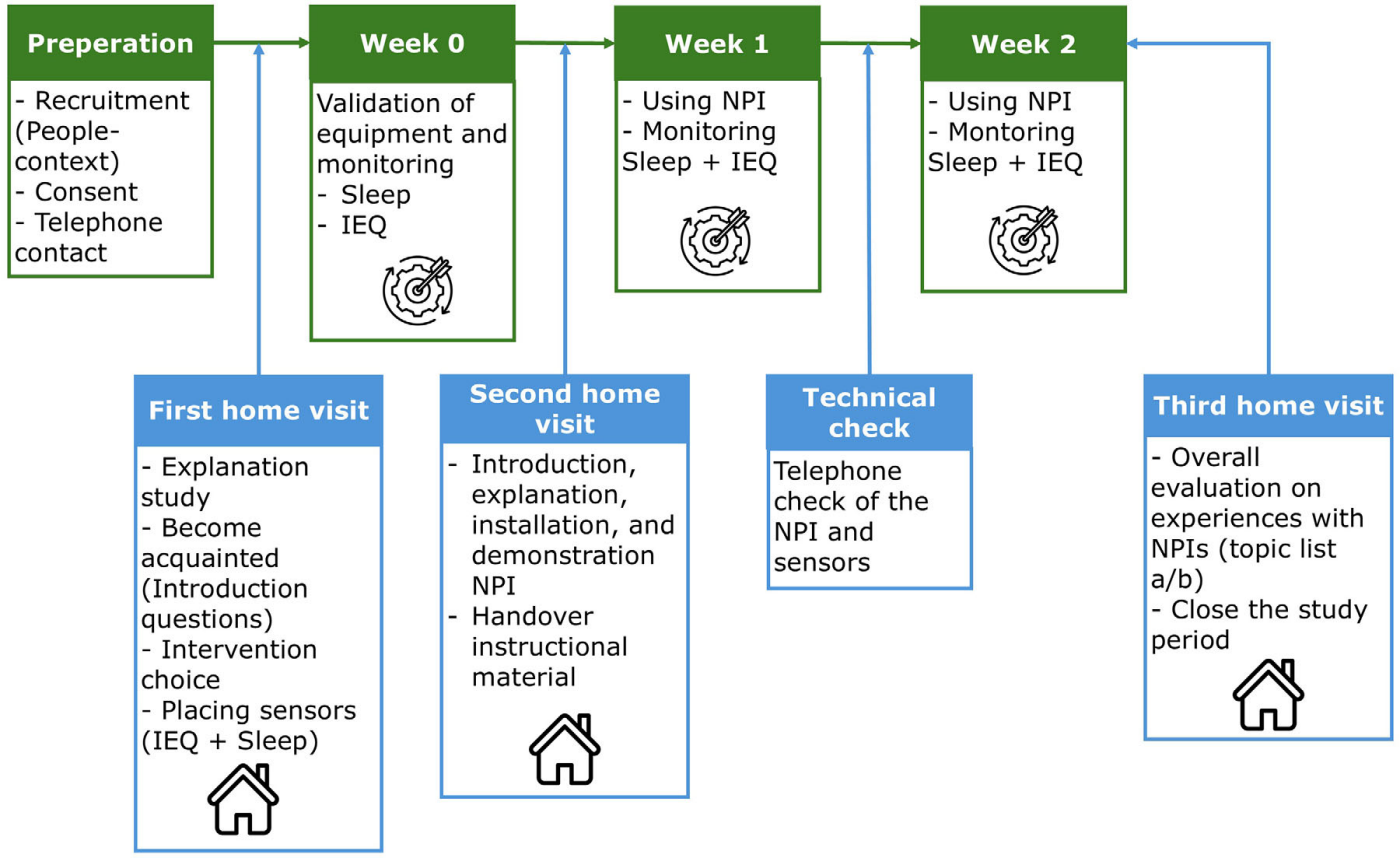
An overview of the pilot study. The blue boxes represent contact moments and the green boxes represent an execution step within the framework. IEQ, indoor environment quality; NPIs, non‐pharmacological interventions

The procedure used to test the framework in the pilot study is presented in Figure 3. It has eight stages. In the preparation phase, participants were recruited through informal caregivers, care and welfare professionals, and by direct and indirect mailing of key figures in the Dutch dementia field, as via local newspapers. Inclusion of participants was based on the criteria described in the DESMEE‐CAP framework (see paragraph People‐context). For the pilot study, the intention was to investigate maximum of four home settings.

In the first home visit, the study was introduced, and the researcher familiarized with the participants. Based on this interaction, appropriate interventions were selected based on the introduction questions (Table 2). Also, the sensors for monitoring sleep and IEQ were installed.

Week 0 was dedicated to determining whether the monitoring equipment works in participants’ homes. In a second home visit the selected interventions and accompanying instructional materials were introduced. These materials include a simplified instruction manual with visual aids. Participants were also invited to contact the helpdesk by phone or email for assistance. An example of the instructional material is provided in Appendix 1.

In Week 1, the participants used the NPIs, while sleep and IEQ were monitored. A technical check was conducted after one week, by phone, to verify the status of the interventions and the functioning of the sensors. In Week 2, participants continued using the interventions, with ongoing monitoring of IEQ and sleep. A third and final home visit was conducted to collect participants’ experiences through an interview guided by a topic list (see Table 3). In parallel, monitoring equipment and the NPIs were collected.

In the pilot study, all results were analyzed and reported in a descriptive manner to identify opportunities to improve the DESMEE‐CAP framework. These opportunities were then discussed with the experts.

## 3 RESULTS

### 3.1 Results, reflections, and adjustments of the framework

The pilot study was carried out between June 2023 and June 2024, involving three participants: two PwD and one informal caregiver. The assessment was focused on experiences concerning the NPIs, the monitoring of sleep and IEQ, and participation in the study, and whether the DESMEE‐CAP framework was able to capture this information in the desired way.

#### 3.1.1 People‐context and home conditions

Recruiting participants for the pilot study proved to be a demanding task, as it involved significant time and effort to locate suitable candidates. A network of individuals who work or live near the target group can be beneficial. Additionally, several factors deterred people from participating, such as existing responsibilities and reluctance to accepts additional commitments. The use of (new) technology also caused uncertainty and doubt. It was concluded, that collaboration with people and organizations in direct contact with the target group, such as healthcare and welfare organizations, is essential. These organizations often have established trust with potential participants and are better able to reach them.^47^

During the recruitment for dyads, it was noticed that PwD living alone wanted to participate. Depending on cognitive capabilities, it was concluded, after consulting the expert, that PwD living alone may also participate in the study. Therefore, the inclusion criteria can be updated allowing people living alone to participate as well. This modification will enlarge the potential target group. Nevertheless, then the cognitive capabilities are more important because the participant living alone needs to understand what is happening at home at any moment. In the pilot study, this was assured by getting acquainted with the PwD, also in consultation with a caregiver. It is regarded helpful that the DESMEE‐CAP framework and the procedure in the pilot study applied several contact moments between participants and the researcher. Weekly contact with the participant is recommended in any case. Another finding from the pilot study is that it is necessary to have a suitable (compatible for the Oura application) smartphone or tablet available for each participant. This is needed to retrieve sleep data. It was stated earlier that at least one device (smartphone or tablet) is available for every household, but this should be changed to one device for each participant within a household, that is, person with dementia and the caregivers.

Participating in the study involves having monitoring devices installed at homes and using NPIs. Although this may not be a major change, it does represent an adjustment in their daily lives. Because of the study's explorative nature and the target group's vulnerability, the framework assumes that people continue their everyday lives during the study. In that way, it is possible to record whether the NPIs fit into people's daily lives and whether they provide support in some way.

#### 3.1.2 Experiences with NPIs

The experiences with the NPIs were collected via a semi‐structured interview (Table 3) at the end of the research period. From the pilot study, it was concluded that the interview gave insight into the participants' experiences using the NPIs and their experiences regarding participation in the research.

However, none of the participants in the pilot used the notebook to document their experiences. According to the procedure of the pilot study (see Figure 3) participants were contacted mid‐way through the study period to check their progress and address any questions or issues. During these phone calls, the researcher took notes. These were relevant to analyze the participants experiences as captured by the researcher. Given the small number of participants (*N* = 3), it was decided to retain the notebook as part of the DESMEE‐CAP framework, though there appears a need to encourage participants to use the notebook.

#### 3.1.3 Monitoring of indoor environment

The objective monitoring of the IEQ is performed with two different sensors, in both rooms (see Table 4). The placement of the sensors should be done in consultation with the participants to avoid inconvenience during the research period. The participants in the pilot study did not experience the sensors as unpleasant, so this can be maintained. It seems important to check the functioning of the sensors regularly (two times a week) during the research period to avoid missing data. Remote access to the sensor data is preferred to avoid extra interference in the daily life of the participants. This should be added to the framework.

#### 3.1.4 Objective sleep monitoring

The objective sleep monitoring was performed using the Oura ring (Gen3). The Oura ring must be used in combination with an application on a smartphone or tablet. This appeared to be an issue in the pilot study, not all participants were familiar with using either a smartphone or tablet. Besides, data synchronization requires some user action (opening the application and ensuring connection to the ring), which sometimes caused difficulties or was forgotten by participants. This led to missing data during the pilot study. Providing a clear and thorough explanation of the ring and its application is essential. Since using this kind of technology is not common, it is important to ensure that participants understand how it works and how it will benefit them. In the pilot study, this ultimately achieved using three different methods: explanation, demonstration, and personal experimentation. These three steps can help alleviate any concerns and increase their comfort with the intervention and the measurements. Another remark related to the Oura ring, is the need to recharge the sensor regularly; people need to be remembered to do this as they are not used to it. It is highly recommended to perform regular (remotely) checks on the web‐based dashboard of the Oura ring to ensure that synchronization is maintained. This helps to ensure that the data collected is consistent and that the technology functions correctly. Regular checks can also help to identify and resolve any issues promptly, ensuring continuity of monitoring.

#### 3.1.5 Subjective sleep measurements

The subjective measurement of sleep is focused only on the informal caregiver and is conducted through a questionnaire. In the pilot study, one informal caregiver participated. Unfortunately, despite the instructions, the responses provided by the informal caregiver were about the person with dementia,. This suggests that the instructions were not clearly understood. Therefore, it is essential to ensure that the instructions are communicated clearly (easy to understand) more than once and to verify that they are understood.

### 3.2 Adjustments of the framework

With the pilot study, it was possible to test the framework. Despite the small sample size, it was possible to improve the framework. Supported by the earlier discussion of the experiences from the pilot study, Table 5 summarizes the changes made to the DESMEE‐CAP framework, along with some key points for future researchers to consider. The core principles of the initial framework remain intact.

**TABLE 5:** Summary of the changes made in the DESMEE‐CAP framework

Aspect	Change / attention points
People‐context	The recruitment can be challenging, so collaboration with people and organizations in direct contact with the target group is necessary. Not only dyads can participate, but also people with dementia who live alone can participate in the study. It is important, however, that people understand what the study is about and what is going to happen. A suitable (compatible with Oura application) smartphone or tablet should be available for each participant. Instead of at least one per household. Having weekly contact with the participants.
Conditions	No changes were made.
Experiences with NPIs	No changes were made.
Objective sleep monitoring	Give a clear explanation of the operation of the Oura Ring (gen. 3) and about the synchronization and the charging. This can be done by: handing over the instructional material (see Appendix 1), giving a demonstration, personal experimentation, and checking if the explanation is understood. Check, on a regular basis, if the data is synchronized. This can be done by checking whether the data has been uploaded on the dashboard.
Subjective sleep measurements	A clear (easy to understand) explanation of the instructions for filling in the questionnaire, and check if information is understood well.
IEQ monitoring	Check the functioning of the sensors twice a week. The sensors preferably should be readable remotely. The check refers to the IEQ data being sent to the dashboard.

Abbreviations: IEQ, indoor environmental quality; NPIs, non‐pharmacological interventions.

## 4 DISCUSSION

The main objective of this study was to develop a framework to capture the experiences with NPIs by community living PwD and their caregivers. In addition, a pilot study is executed to validate the DESMEE‐CAP framework, that provides insight into the sleep and sleep conditions of PwD and their caregivers at home when exploring participants’ experiences with NPIs that support sleep. The framework was developed by examining methods from previous studies that investigated sleep quality and sleep strategies. However, earlier studies focused on healthy people or on other parameters For example the impact of the thermal environment and air quality on biological rhythms (e.g.,^48^). The DESMEE‐CAP framework combines (1) gathering experiences with the use of NPIs, (2) monitoring objective and subjective sleep, and (3) monitoring IEQ. The latter is a novel approach in monitoring PwD and their caregivers. Until, now environmental parameters are separately monitored and not in a holistic matter with usually a focus on light exposure or light therapy (e.g.^49^). The framework use existing questionnaires as starting point, such as the USE and CSI questionnaires, and closely follows the protocol described by Mishra et al.^37^ and used by Van der Veen.^32^

The validity of the DESMEE‐CAP framework was tested in a pilot study with the target group. That study confirmed that the DESMEE‐CAP framework is applicable and valid for use at contexts that aims to gain experiences of NPIs. Content validity is ensured by using literature and expert input during the frameworks’ development. Face validity is assessed by using the framework in practice and evaluating it with researchers and participants during interviews. The pilot study confirmed that the framework includes all essential aspects to examine experiences and monitor sleep and indoor environmental parameters, supporting its content validity. Although some adjustments were needed (Table 5) these mainly concerned instructions and regularly checking sensors and data gathering.

Dewing^50^ proposes using simplified information down to single keywords with or without pictures, to pass over information. Using the principles of Errorless Learning (EL) could help improve providing the instructions to the participants in this framework. EL is effective in learning activities of daily living for PwD.^51^ The principles of EL include breaking tasks into smaller steps, immediate error correction, encouraging participants not to guess, illustrating task steps, fading cues and prompts when steps are successfully performed, and repeating instructions at increasing time intervals.^52^

In developing the framework, it was crucial to consider the vulnerability of the target group, community‐dwelling PwD and their caregivers. The challenges posed by dementia for both the individuals and their caregivers were carefully considered. Additionally, recognizing that PwD are diverse, research approaches should be tailored to participants and their (home) context, as mentioned by Novek and Wilkinson^53^ in their review.

Given the vulnerability of the group, major changes in the daily lives of participants should be avoided because this may increase the strain on informal caregivers and confuse the person with dementia, affecting the intervention's outcome. Measuring the effect of NPIs is not straightforward because many contextual factors affect good sleep. A more extended research period may provide better conditions to assess the effect of NPIs. This study focuses on the experiences with the NPIs. The DESMEE‐CAP framework presents no limitation to the study period, though the burden on the PwD and caregivers should be considered and monitored closely if the experiences with the NPIs are negative.

One factor that may influence experiences with NPIs is the target group. An NPI that is not focused on PwD may not adequately match their abilities and needs. Of the NPIs selected for the pilot study, not all were designed with a focus on PwD. TimeSteps (supporting maintaining daily rhythm and transition from day to night) and Qwiek.snooze (supporting falling asleep and sleep through) are explicitly designed for people with dementia. Similarly, Lizz (supporting maintaining daily rhythm and transition from day to night) is adapted for PwD. However, Somnox 2 (supporting falling asleep and sleep through) and the weighted blanket (supporting falling asleep and sleep through) are not intended for any specific target group. This demonstrates that NPIs, even those not specifically developed for this group, can be explored in studies examining NPIs effects, using the DESMEE‐CAP framework. In this way interventions, can be aligned with challenges and experiences of PwD and caregivers regarding sleep and with the strategies to tackle sleep issues.^23^

In the pilot study the DESMEE‐CAP framework appears to be a valid and practical method for examining the experience of community‐dwelling people with dementia and their caregivers in using NPIs to support sleep. Additionally, it enables researchers to monitor both sleep and IEQ. The DESMEE‐CAP was pilot‐tested with the intended target group and demonstrated its potential to contribute to the development of appropriate sleep support strategies for people with dementia and their caregivers. While the framework proved to be usable, some modifications were made to optimize the framework.

### 4.1 Limitations

The DESMEE‐CAP framework was assessed, and experiences were examined over a two‐week period. This is a relatively short period, and participants may need more time to get acquainted and fit the NPIs into their daily lives. As health‐related habits can start forming within about 2 months, but that the required time varies across individuals.^54^ Additionally, sleep can be influenced by several factors; people may occasionally experience fewer or more sleep problem than usual. The framework allows studies with a more extended research period. The study design though (see Figure 3) should be adjusted regarding the timeline. Recruitment of participants was complex, with several factors deterring people from participating in the pilot study. Various strategies should be used to recruit participants.^47^ The framework was tested in a pilot study with a small sample (*N* = 3), including two PwD and one informal caregiver. While it was possible to assess the use of the framework in a real‐life setting, the limited sample size may restrict the generalizability of the findings for the framework. Due to the group's heterogeneity, it may be necessary to adapt the framework. What worked in this small group may not work for other samples. As the framework and study approach are flexible, modifications though can be made to fit the context.

Determining the effectiveness of NPIs is currently not feasible with the framework as no evaluation items that determine the NPIs’ effectiveness have been defined. This was also not the intention of the study. Future studies could address this topic. The DESMEE‐CAP framework can serve as an initial assessment to select NPIs with good potential, based on assessed experiences. For analyzing the NPIs effectiveness, it is important to measure sleep in a validated (gold standard) way for a more extended period. The gold standard for measuring sleep in people with sleep disorders is polysomnography.^55^ It is unclear whether this applies to people with dementia, and it is questionable whether it is ethical to use polysomnography. Instead, monitoring sleep latency, awakenings, wake after sleep onset, and sleep efficiency may be an alternative way to monitor sleep in people with dementia. Monitoring IEQ was realized as expected though the light measurement was limited to measuring light in lux (visual effect) and not in a metric expressing non‐visual effects. Due to the limitation of the light sensor.

### 4.2 Future studies

The framework is focused on experiences with NPIs to support sleep, the NPIs can vary in future studies. A previous study presented different NPIs^56^ aiming to contribute to lower the care burden and/or improve sleep quality. The framework gives guidance to examine those NPIs as well. In case they consider the same themes and strategies that underline the here developed framework. Relevance of specific NPIs depends on the needs and capabilities of the participants. Future users of the framework should select the appropriate NPIs for their study context and participants. User experiences can be influenced by someone's digital capabilities. It is known that the older generation is less well able to use technology due to a lack of digital knowledge and skills (e.g.^57^). However, other studies do mention that digital knowledge, experience, and skills of older adults can be trained.^58^

## AUTHOR CONTRIBUTIONS

C.H. and H.K. designed the framework. HK supervision writing and editing. ML supervision, writing and editing. C.H. data collection, writing and editing. All authors discussed the results and contributed to the final manuscript.

## CONFLICT OF INTEREST STATEMENT

All authors declare that they have no conflicts of interest. the authors has a conflict of interest to declare. Author disclosures are available in the Supporting Information.

## CONSENT STATEMENT

The Health Domain Research Ethics Committee at Utrecht University of Applied Sciences approved the study protocol of the pilot study (ECO‐GD–ref. 188‐000‐2022). All participants provided their informed consent before the start of the pilot study.

## Supporting information



Supporting Information

Supporting Information

## Data Availability

The data that support the findings of this study are not publicly available due to privacy concerns and the context‐dependent nature of the data. Requests for access to the data should be directed to the corresponding author.
