# Supplementary material for: Developing a structured framework to explore the experiences of people with dementia and their caregivers regarding non‐pharmacological sleep interventions
Source: Alzheimers Dement. 2026 Feb 7;22(2):e71081. doi: 10.1002/alz.71081 (PMC12882557; doi:10.1002/alz.71081)
Supplement: Supplementary file 1 — Supporting Information [file ALZ-22-e71081-s002.pdf]

# ICMJE DISCLOSURE FORM

| | |
|---|---|
| **Date:** | 11/7/2025 |
| **Your Name:** | Chantal Huisman |
| **Manuscript Title:** | DEveloping a Structured FraMework to Explore the Experiences of People with Dementia and their CAregivers regarding non-Pharmacological Sleep Interventions |
| **Manuscript Number (if known):** | ADJ-D-25-01670 |

In the interest of transparency, we ask you to disclose all relationships/activities/interests listed below that are related to the content of your manuscript. "Related" means any relation with for-profit or not-for-profit third parties whose interests may be affected by the content of the manuscript. Disclosure represents a commitment to transparency and does not necessarily indicate a bias. If you are in doubt about whether to list a relationship/activity/interest, it is preferable that you do so.

The author's relationships/activities/interests should be defined broadly. For example, if your manuscript pertains to the epidemiology of hypertension, you should declare all relationships with manufacturers of antihypertensive medication, even if that medication is not mentioned in the manuscript.

In item #1 below, report all support for the work reported in this manuscript without time limit. For all other items, the time frame for disclosure is the past 36 months.

| | | **Name all entities with whom you have this relationship or indicate none (add rows as needed)** | **Specifications/Comments (e.g., if payments were made to you or to your institution)** |
|---|---|---|---|
| | | Time frame: Since the initial planning of the work | |
| 1 | All support for the present manuscript (e.g., funding, provision of study materials, medical writing, article processing charges, etc.) **No time limit for this item.** | ☐ None | |
| | | Regieorgaaan SIA | To institution |
| | | Health~Holland | To institution |
| | | Lizz sleep coach | Participation in the Goede nacht, betere dag" project (Good Night, for a Better Day project Health~Holland, Top Sector Life Sciences & Health, to stimulate public-private partnerships (EXZ.EXZ.01.005). |
| | | Somnox 2 | As above |
| | | Qwiek.snooze | As above |
| | | Dr. Mirjam van Tilborg | Colleague at Utrecht University of Applied Science (voluntary) |
| | | Time frame: past 36 months | |
| 2 | Grants or contracts from any entity (if not indicated in item #1 above). | ☒ **None** | |
| | | Digital Resources supporting sleep of persons living with dEmentia while Alleviating caregivers' burden (DREAm) - HH-PPS-24033-SPR | Participation in DREAm project (Health~Holland, Top Sector Life Sciences & Health) |
| | | | |
| | | | |

| | | Name all entities with whom you have this relationship or indicate none (add rows as needed) | Specifications/Comments (e.g., if payments were made to you or to your institution) |
|---|---|---|---|
| **3** | Royalties or licenses | ☒ **None** | |
| **4** | Consulting fees | ☒ **None** | |
| **5** | Payment or honoraria for lectures, presentations, speakers bureaus, manuscript writing or educational events | ☒ **None** | |
| **6** | Payment for expert testimony | ☒ **None** | |
| **7** | Support for attending meetings and/or travel | ☒ **None** | |
| **8** | Patents planned, issued or pending | ☒ **None** | |
| **9** | Participation on a Data Safety Monitoring Board or Advisory Board | ☒ **None** | |
| **10** | Leadership or fiduciary role in other board, | ☒ **None** | |

| | | Name all entities with whom you have this relationship or indicate none (add rows as needed) | Specifications/Comments (e.g., if payments were made to you or to your institution) |
|---|---|---|---|
| | society, committee or advocacy group, paid or unpaid | | |
| **11** | Stock or stock options | ☒ **None** | |
| **12** | Receipt of equipment, materials, drugs, medical writing, gifts or other services | ☒ **None** | |
| **13** | Other financial or non-financial interests | ☒ **None** | |

**Please place an "X" next to the following statement to indicate your agreement:**

☒ I certify that I have answered every question and have not altered the wording of any of the questions on this form.

# ICMJE DISCLOSURE FORM

**Date:** November 10, 2025

**Your Name:** Helianthe Kort

**Manuscript Title:** DEveloping a Structured FraMework to Explore the Experiences of People with Dementia and their 3 CAregivers regarding non-Pharmacological Sleep Interventions

**Manuscript Number (if known):** Click or tap here to enter text.

In the interest of transparency, we ask you to disclose all relationships/activities/interests listed below that are related to the content of your manuscript. "Related" means any relation with for-profit or not-for-profit third parties whose interests may be affected by the content of the manuscript. Disclosure represents a commitment to transparency and does not necessarily indicate a bias. If you are in doubt about whether to list a relationship/activity/interest, it is preferable that you do so.

The author's relationships/activities/interests should be defined broadly. For example, if your manuscript pertains to the epidemiology of hypertension, you should declare all relationships with manufacturers of antihypertensive medication, even if that medication is not mentioned in the manuscript.

In item #1 below, report all support for the work reported in this manuscript without time limit. For all other items, the time frame for disclosure is the past 36 months.

| | | **Name all entities with whom you have this relationship or indicate none (add rows as needed)** | **Specifications/Comments (e.g., if payments were made to you or to your institution)** |
|---|---|---|---|
| | | Time frame: Since the initial planning of the work | |
| 1 | All support for the present manuscript (e.g., funding, provision of study materials, medical writing, article processing charges, etc.) **No time limit for this item.** | ☐ **None** | |
| | | Lizz sleep coach | Participation in the Goede nacht, betere dag" project (Good Night, for a Better Day project Health~Holland, Top Sector Life Sciences & Health, to stimulate public-private partnerships (EXZ.EXZ.01.005). |
| | | TimeSteps | As above |
| | | Somnox 2 | As above |
| | | Qwiek.snooze | As above |
| | | Dr. Mirjam van Tilborg | Colleague at Utrecht University of Applied Science (voluntary) |
| | | Dr. Marcel Loomans | Co-author and colleague at Eindhoven University of Technology (Normal salary) |
| | | Msc Chantal Huisman | PhD candidate at Eindhoven University of Technology and Colleague at Utrecht University of Applied Science (Normal salary) |
| | | Time frame: past 36 months | |
| **2** | Grants or contracts from any entity (if not indicated in item #1 above). | ☒ **None** | |
| | | Digital Resources supporting sleep of persons living with dEmentia while Alleviating caregivers' burden (DREAm) - HH-PPS-24033-SPR | Participation in DREAm project (Health~Holland, Top Sector Life Sciences & Health) |
| | | | |
| | | | |

| | | Name all entities with whom you have this relationship or indicate none (add rows as needed) | Specifications/Comments (e.g., if payments were made to you or to your institution) |
|---|---|---|---|
| **3** | Royalties or licenses | ☒ **None** | |
| | | | |
| | | | |
| | | | |
| **4** | Consulting fees | ☒ **None** | |
| | | | |
| | | | |
| | | | |
| | | | |
| **5** | Payment or honoraria for lectures, presentations, speakers bureaus, manuscript writing or educational events | ☒ **None** | |
| | | | |
| | | | |
| | | | |
| **6** | Payment for expert testimony | ☒ **None** | |
| | | | |
| | | | |
| | | | |
| **7** | Support for attending meetings and/or travel | ☒ **None** | |
| | | | |
| | | | |
| | | | |
| **8** | Patents planned, issued or pending | ☒ **None** | |
| | | | |
| | | | |
| | | | |
| **9** | Participation on a Data Safety Monitoring Board or Advisory Board | ☒ **None** | |
| | | | |
| | | | |
| | | | |
| **10** | Leadership or fiduciary role in other board, | ☒ **None** | |
| | | | |

| | | Name all entities with whom you have this relationship or indicate none (add rows as needed) | Specifications/Comments (e.g., if payments were made to you or to your institution) |
|---|---|---|---|
| | society, committee or advocacy group, paid or unpaid | | |
| **11** | Stock or stock options | ☒ **None** | |
| **12** | Receipt of equipment, materials, drugs, medical writing, gifts or other services | ☒ **None** | |
| **13** | Other financial or non-financial interests | ☒ **None** | |

**Please place an "X" next to the following statement to indicate your agreement:**

☒ I certify that I have answered every question and have not altered the wording of any of the questions on this form.

# ICMJE DISCLOSURE FORM

**Date:** 11/10/2025

**Your Name:** M.G.L.C. Loomans

**Manuscript Title:** DEveloping a Structured FraMework to Explore the Experiences of People with Dementia and their 3 CAregivers regarding non-Pharmacological Sleep Interventions

**Manuscript Number (if known):** Click or tap here to enter text.

In the interest of transparency, we ask you to disclose all relationships/activities/interests listed below that are related to the content of your manuscript. "Related" means any relation with for-profit or not-for-profit third parties whose interests may be affected by the content of the manuscript. Disclosure represents a commitment to transparency and does not necessarily indicate a bias. If you are in doubt about whether to list a relationship/activity/interest, it is preferable that you do so.

The author's relationships/activities/interests should be defined broadly. For example, if your manuscript pertains to the epidemiology of hypertension, you should declare all relationships with manufacturers of antihypertensive medication, even if that medication is not mentioned in the manuscript.

In item #1 below, report all support for the work reported in this manuscript without time limit. For all other items, the time frame for disclosure is the past 36 months.

| | | Name all entities with whom you have this relationship or indicate none (add rows as needed) | Specifications/Comments (e.g., if payments were made to you or to your institution) |
|---|---|---|---|
| | | **Time frame: Since the initial planning of the work** | |
| 1 | All support for the present manuscript (e.g., funding, provision of study materials, medical writing, article processing charges, etc.) **No time limit for this item.** | ☐ **None** | |
| | | Lizz sleep coach | Participation in the Goede nacht, betere dag" project (Good Night, for a Better Day project Health~Holland, Top Sector Life Sciences & Health, to stimulate public-private partnerships (EXZ.EXZ.01.005). |
| | | TimeSteps | As above |
| | | Somnox 2 | As above |
| | | Qwiek.snooze | As above |
| | | Dr. Mirjam van Tilborg | Colleague at Utrecht University of Applied Science (voluntary) |
| | | **Time frame: past 36 months** | |
| **2** | Grants or contracts from any entity (if not indicated in item #1 above). | ☐ **None** | |
| | | NWO (MIST – lead Twente University) | Research grant related to research on ventilation – air cleaning and exposure to pathogens |
| | | Health Holland (CLAIRE – lead Utrecht University) | Research grant related to research on portable air cleaning devices for application in classrooms |
| | | Government (P3Venti – lead TNO) | Research grant related to research on ventilation in the context of pathogen exposure |
| | | Interreg Government (Healthy Building Movement – lead Venlo municipality) | Research grant related to research on healthy buildings |

| | | Name all entities with whom you have this relationship or indicate none (add rows as needed) | Specifications/Comments (e.g., if payments were made to you or to your institution) |
|---|---|---|---|
| | | Health Holland (DREAm – lead Utrecht University of Applied Sciences) | Research grant related to IEQ and sleep of people with dementia (manuscript is precursor to this project) |
| | | RVO (government) | Travel grant for participating in IEA Annex 86 |
| | | Binnenklimaat Nederland | Grant for developing Program of Requirements Healthy indoor climate Longterm care facilities |
| | | Digital Resources supporting sleep of persons living with dEmentia while Alleviating caregivers' burden (DREAm) - HH-PPS-24033-SPR | Participation in DREAm project (Health~Holland, Top Sector Life Sciences & Health) |
| **3** | Royalties or licenses | ☒ **None** | |
| **4** | Consulting fees | ☒ **None** | |
| **5** | Payment or honoraria for lectures, presentations, speakers bureaus, manuscript writing or educational events | ☒ **None** | |
| **6** | Payment for expert testimony | ☒ **None** | |
| **7** | Support for attending meetings and/or travel | ☒ **None** | |

| | | Name all entities with whom you have this relationship or indicate none (add rows as needed) | Specifications/Comments (e.g., if payments were made to you or to your institution) |
|---|---|---|---|
| **8** | Patents planned, issued or pending | ☒ **None** | |
| | | | |
| | | | |
| | | | |
| **9** | Participation on a Data Safety Monitoring Board or Advisory Board | ☐ **None** | |
| | | HumanIC Project (EU-project) | International Advisory Board |
| | | | |
| | | | |
| **10** | Leadership or fiduciary role in other board, society, committee or advocacy group, paid or unpaid | ☒ **None** | |
| | | | |
| | | | |
| | | | |
| **11** | Stock or stock options | ☒ **None** | |
| | | | |
| | | | |
| | | | |
| **12** | Receipt of equipment, materials, drugs, medical writing, gifts or other services | ☒ **None** | |
| | | | |
| | | | |
| | | | |
| **13** | Other financial or non-financial interests | ☒ **None** | |
| | | | |
| | | | |
| | | | |

**Please place an "X" next to the following statement to indicate your agreement:**

☒ I certify that I have answered every question and have not altered the wording of any of the questions on this form.
