# Supplementary material for: Developing a structured framework to explore the experiences of people with dementia and their caregivers regarding non‐pharmacological sleep interventions
Source: Alzheimers Dement. 2026 Feb 7;22(2):e71081. doi: 10.1002/alz.71081 (PMC12882557; doi:10.1002/alz.71081)
Supplement: Supplementary file 2 — Supporting Information [file ALZ-22-e71081-s001.pdf]

Appendix 1 Example of the instructional material

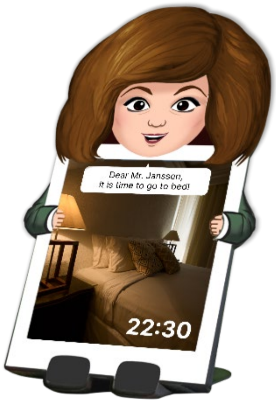

Lizz sleep coach. (source ConnectedCare)

Lizz

Lizz provides you with reminders throughout the day. Place Lizz in a location where you can hear and see her for most of the day. You do not need to move Lizz.

Getting Started

- Lizz must always be plugged into a power socket.
- If the screen is black, press the power button at the top right-hand corner.
- Swipe upwards on the screen with your finger. Tap the "Lizz 2.0" icon at the bottom of the home screen.

Let's Communicate

During the day, Lizz will speak messages encouraging activities, such as taking a walk, opening a window, or going to bed. To hear a message again, tap the speech bubble.

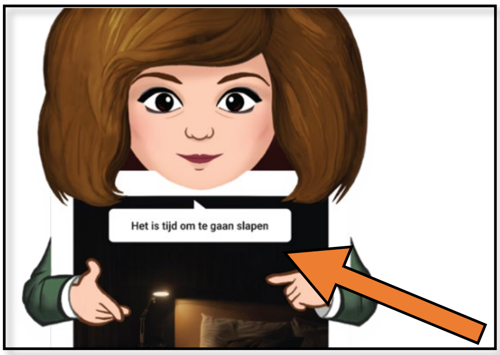

Tap the speech bubble to replay a message

You can answer a question by tapping one of the options at the bottom: **Yes** or **No**.

Keep Lizz Updated

If you want to let Lizz know something about yourself, you can always press the **+** button above the date. For example, you can let her know how you are feeling.

Muting and Sleeping

If you want to mute Lizz during the day, press the speaker icon in the bottom-right corner.

Press the **zzz** button when you go to bed; Lizz will remain silent until you tap the '**wake up**' button.

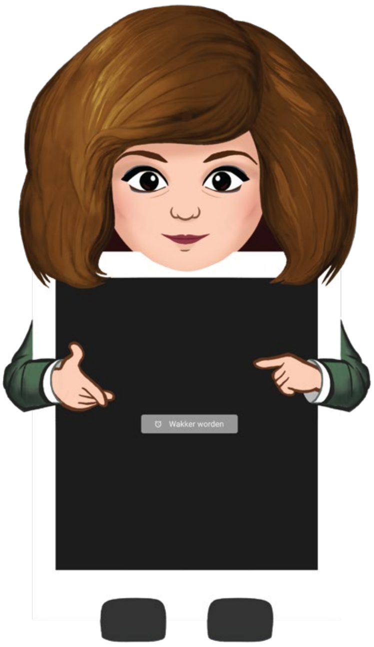

Press 'wake up' to reactivate Lizz

Troubleshooting

If Lizz does not work as expected and you need assistance, contact Chantal Huisman (chantal.huisman@hu.nl / 06 3876 3613). She is available during office hours.

Appendix 2 Non-pharmacological interventions

The four strategies used in the DESMEE-CAP approach are : 1) support keeping a daily rhythm, 2) support the transition from day to night, 3) support falling asleep, and 4) sleep through (see Table 2). The non-pharmacological interventions are selected based on these strategies.

Two possible main non-pharmacological interventions and three optional non-pharmacological interventions were selected for the pilot study, these are presented below.

Non-pharmacological interventions

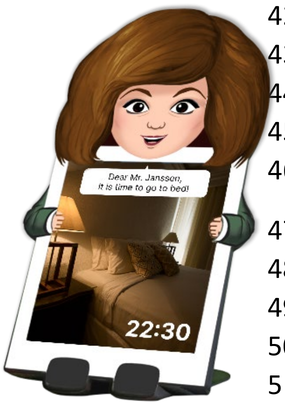

Figure A Lizz sleep coach. (source ConnectedCare)

Two non-pharmacological interventions are selected to support daily rhythm (strategy 1) and support the transition from day to night (strategy 2), 1) Sleep coach Lizz and 2) User-friendly agenda TimeSteps. Each dyad will work with one of these interventions (random, based on wishes and needs). Descriptions of these interventions are provided below.

The Lizz sleep coach (Figure A) is developed as a digital assistant and companion. The assistant can provide reminders, collect self-reports, provide background information, and provide social interaction. It is possible to customise Lizz to fit a person's daily life. Lizz can support somebody to maintain a certain daily rhythm (Strategy 1) by giving reminders, but she can also remind people to go for a walk or go to bed (strategy 2), or she can play music or an audiobook, for example.

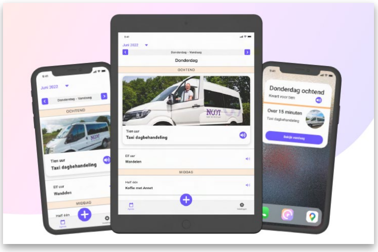
Figure B TimeSteps app. (source: TimeSteps)

TimeSteps is an application (smartphone/tablet) (Figure B) combined with three Philips HUE lights (strategy 1 and 2). The TimeSteps application is developed to support people with dementia with time orientation by providing written time and part of the day. In addition, the application can give users reminders to support their daily rhythms. An informal caregiver can set this reminder, which can be spoken by the application. In this study, the TimeSteps application is combined with Philips HUE lights (smart light) to provide a light cue in addition to a spoken reminder to encourage people to follow up on the reminder. For example, the TimeSteps application reminds a person about the time to go to bed, at the same time, the Philips HUE light in the bedroom will turn on with a maximum of 300 lux (support transition day to night).

There are three other non-pharmacological interventions: 1) Somnox, 2) Qwiek.snooze, and 3) weighted blanket. Each participant or duo will work with one of these interventions (in consultation). Descriptions of these interventions are provided below.

Somnox 2 (Figure 5) is developed as a sleeping aid that provides tactile pressure stimulation of a breathing pattern. This may help the user relax by feeling a simulated breathing pattern on the chest. Somnox also provides calming sounds to help soothe and support falling asleep (Strategies 3 and 4) [46].

Qwiek.snooze (Figure 6) is a smart music pillow designed to support people with dementia with their sleep. By using relaxing music, the Qwiek.snooze supports falling asleep and sleeping through the night (strategies 3 and 4) [47].

A weighted blanket (Figure E) is another non-pharmacological intervention used in this study. A study by [NO_PRINTED_FORM] [48] show a tentative positive effect on the release of melatonin at bedtime when a weighted blanket is used by young, healthy adults (strategies 3 and 4).

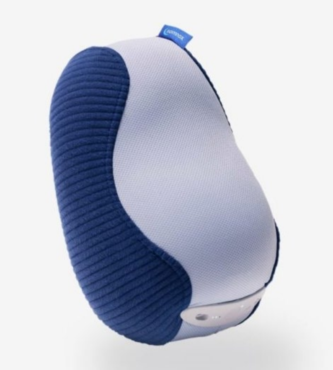

Figure C Sonmox 2 (source: Somnox)

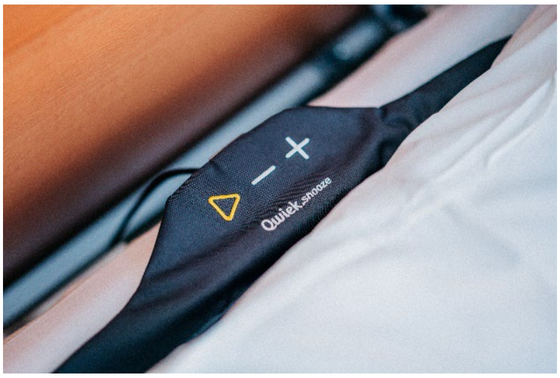

Figure D Qwiek.snooze (source: Qwiek bv)

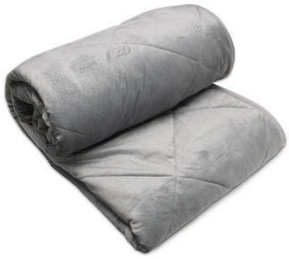

Figure E Weigthed Blanket (source: Lucovitaal)

Lizz and TimeSteps can support people with dementia to keep their rhythm during the day. TimeSteps en Lizz also supports the transition from day to night. Stimulate/motivate to prepare to go to bed and fall asleep easily. The other non-pharmacological interventions are intended for use in the bed. These interventions are selected because they may support falling asleep or sleeping through. An overview of the non-pharmacological interventions can be seen in Table X.

Table X. Overview of strategies with the matching product

**Strategy**	**Product**
Strategy 1 and 2	Lizz<br>- Stand-alone tablet with faceplate<br>- Paired with caregiver (in development)<br>- Agenda with visual and spoken reminders<br>- Support transition day to night<br>- Collecting self-reports
Strategy 1 and 2	TimeSteps<br>- Application<br>- Paired with caregiver<br>- Time orientation<br>- Agenda with visual and spoken reminders
Strategy 3 and 4	Somnox<br>- Sleeping aid<br>- Falling asleep
Strategy 3 and 4	Qwiek.snooze<br>- Music pillow<br>- Falling asleep<br>- Sleeping through
Strategy 3 and 4	Weighted blanket<br>- Blanket<br>- Falling asleep<br>- Sleeping through

88
